# The effects of vitamin C supplementation on pre-eclampsia in Mulago Hospital, Kampala, Uganda: a randomized placebo controlled clinical trial

**DOI:** 10.1186/1471-2393-14-283

**Published:** 2014-08-21

**Authors:** Paul Kiondo, Gakenia Wamuyu-Maina, Julius Wandabwa, Gabriel S Bimenya, Nazarius Mbona Tumwesigye, Pius Okong

**Affiliations:** Department of Obstetrics and Gynaecology, School of Medicine, Makerere University College of Health Sciences, P.O Box 7072, Kampala, Uganda; School of Public Health, Makerere University College of Health Sciences, P.O Box 7072, Kampala, Uganda; Department of Obstetrics and Gynaecology, Walter Sisulu University, Private Bag X1, Mthatha, 5117 South Africa; Department of Pathology, School of Medicine, Makerere University College of Health Sciences, P.O Box 7072, Kampala, Uganda; Department of Reproductive Health, Uganda Christian University, P.O Box 4, Mukono, Uganda

## Abstract

**Background:**

Oxidative stress plays a role in the pathogenesis of pre-eclampsia. Supplementing women with antioxidants during pregnancy may reduce oxidative stress and thereby prevent or delay the onset pre-eclampsia. The objective of this study was to evaluate the effect of supplementing vitamin C in pregnancy on the incidence of pre-eclampsia, at Mulago hospital, Kampala, Uganda.

**Methods:**

This was a (parallel, balanced randomization, 1:1) placebo randomized controlled trial conducted at Mulago hospital, Department of Obstetrics and Gynecology. Participants included in this study were pregnant women aged 15-42 years, who lived 15 km or less from the hospital with gestational ages between 12-22 weeks. The women were randomized to take 1000mg of vitamin C (as ascorbic acid) or a placebo daily until they delivered. The primary outcome was pre-eclamsia. Secondary outcomes were: severe pre-eclampsia, gestational hypertension, preterm delivery, low birth weight and still birth delivery. Participants were 932 pregnant women randomized into one of the two treatment arms in a ratio of 1:1. The participants, the care providers and those assessing the outcomes were blinded to the study allocation.

**Results:**

Of the 932 women recruited; 466 were randomized to the vitamin and 466 to the placebo group. Recruitment of participants was from November 2011 to June 2012 and follow up was up to January 2013. Outcome data was available 415 women in the vitamin group and 418 women in the placebo group.

There were no differences in vitamin and placebo groups in the incidence of pre-eclampsia (3.1% versus 4.1%; RR 0.77; 95% CI: 0.37-1.56), severe pre-eclampsia (1.2% versus 1.0%; RR 1.25; 95% CI: 0.34-4.65), gestational hypertension(7.7% versus 11.5%; RR 0.67; 95% CI: 0.43-1.03), preterm delivery (11.3% versus 12.2%; RR 0.92; 95% CI: 0.63-1.34), low birth weight (11.1% versus 10.3%; RR 1.07; 95% CI: 0.72-1.59) and still birth delivery (4.6% versus 4.5%; RR 1.01; 95% CI: 0.54-1.87).

**Conclusions:**

Supplementation with vitamin C did not reduce the incidence of pre-eclampsia nor did it reduce the adverse maternal or neonatal outcomes. We do not recommend the use of vitamin C in pregnancy to prevent pre-eclampsia.

**Trial registration:**

This study was registered at the Pan African Clinical Trial Registry, PACTR201210000418271 on 25^th^ October 2012.

## Background

Hypertensive disorders in pregnancy are a leading cause of maternal mortality worldwide [1] especially in low resource settings [2]. Pre-eclampsia, defined by hypertension and proteinuria, is a multisystem disorder which is associated with, neurological disturbances, haematological disturbances, liver disease, and abnormal renal function [3, 4]. Pre-eclampsia affects 2-10% of all pregnancies and may be higher in low resource settings [5, 6].

Severe pre-eclampsia may result in serious complications like eclampsia and the HELLP syndrome although these complications are rare. In a study conducted in Mulago hospital, pre-eclampsia/eclampsia contributed to 12% maternal deaths among women admitted with severe maternal morbidity [7]. Pre-eclampsia contributes to neonatal morbidity and mortality. Pre-eclampsia contributed to 5.4% perinatal deaths and 24% of intrauterine growth restriction [8] and is associated with 25.8% of preterm births [9].Small-for-gestational age babies are associated with health risks later in life like obesity and insulin resistance, which may manifest as hypertension and diabetes [10]. Preterm delivery is associated with neonatal and infant mortality. Preterm infants are more prone to respiratory complications like respiratory distress syndrome, transient tachypnea of the new born and persistent pulmonary hypertension [11].

Antioxidants are essential in maintaining cellular integrity in normal pregnancy by reducing lipid peroxidation reactions. Thus they protect proteins, enzymes, and cells from destruction by peroxides. Antioxidant defense mechanisms include intracellular and extracellular enzymes (viz. catalase, superoxide dismutase, glutathione peroxidase), free radical scavengers (vitamin C & E, caretenoids, glutathione and serum albumin) and metabolites (bilirubin and uric acid) [12].

The aetiology of pre-eclampsia is unknown. There is incomplete trophoblast invasion of the spiral arteries [13] which lead to abnormal placental development and, placental hypo perfusion and ischemia. Women with pre-eclampsia have increased oxidative stress [14], increased markers of oxidative stress like, 8-iso-prostaglandinF_2α,_ lipid peroxides [15] and, have low plasma and placental concentration of antioxidants [12, 16]. This has led to the hypothesis that placental hypo perfusion may promote a state of oxidative stress in which there is a release of factors to the maternal circulation [13, 17]. The factors include, lipid peroxides, cytokines and syncitiotrophoblast microvillus fragments [18] which are highly reactive molecules that consume the antioxidants.

Oxidative stress combined with an exaggerated inflammatory response may result in the release the maternal factors that cause endothelial dysfunction [18]. Endothelial cell dysfunction is responsible for the clinical signs of pre-eclampsia like hypertension and protienuria. Response to oxidative stress depends on availability of low density lipoproteins, genetic predisposition, immune mal-adaptation [19] and dietary deficiency of antioxidants [20]. Supplementation with antioxidants may modify the women’s response to oxidative stress and therefore limit the systemic and utero-placental endothelial damage observed in pre-eclampsia.

Vitamin C scavenges free radicals in aqueous solution and may have a role in the management of pre-eclampsia. Studies show that vitamin C may be protective against development of pre-eclampsia [21, 22]. Supplementation with vitamin C and E reduces oxidative stress and endothelial dysfunction [23] and, pre-eclampsia [24]. However, subsequent studies have not confirmed this [25–27]. Most of these studies were conducted in high resource settings where the nutritional status of the women is high.

The purpose of this study therefore was to evaluate the effects of vitamin C supplementation on the occurrence of pre-eclampsia in a low resource setting where the nutritional status of the women is low and most pregnant women are deficient in vitamin C [28].

## Methods

### Trial design

This was a (parallel, balanced randomization, 1:1,) placebo controlled trial conducted from November 2011 to January 2013, in Uganda.

### Participants

Participants included in this study were pregnant women aged 15-42 years, lived 15 km or less from the hospital with gestational ages between 12-22 weeks. Women were excluded if they had hypertension, renal diseases or diabetes mellitus, were taking vitamin C supplements of >200 mg/day or had contraindications to vitamin C.

### Setting

This study was conducted at Mulago Hospital, Department of Obstetrics and Gynaecology. Mulago Hospital is a National Referral Hospital for Uganda and a Teaching Hospital for Makerere University College of Health Sciences. In Uganda about 90% of the women attend antenatal clinic at least once during pregnancy. Mulago hospital delivers about 22,000 women annually and about 50,000 women attend antenatal clinic in 1 year.

### Interventions

The women were randomized in one of the two treatment arms using computer generated random numbers in a ratio of 1:1. The women allocated to the vitamin arm were advised to take a tablet of 1000 mg of vitamin C (as ascorbic acid) daily until they delivered. The women allocated to the placebo arm were advised to take a placebo tablet (micro-crystalline cellulose) daily which was identical to the vitamin tablets in shape, color and size. They were to swallow the tablets daily and leave the unused tablets.

### Study procedures

Eligible women were identified from the antenatal clinic by research assistants who were trained midwives. The women were conducted through an informed consent procedure and gave a written informed consent. The women were interviewed about their socio-demographic characteristics, medical and family history, and their present and past obstetric performances. A thorough clinical and obstetrical examination was done, weight was taken and height measured. A mid upper arm circumference was measured by a trained assistant. Blood was drawn for complete blood counts, renal and liver function tests and HIV screening.

### Follow up

All the women were followed until they delivered. The study participants received care according the Uganda Ministry of Health guidelines. Routine visits were done by the research team every four weeks until the women delivered. If a woman missed the scheduled visit she was contacted by telephone and visited at her home and, if necessary was transported to hospital for evaluation by the study physicians. During the routine assessments the women were asked about compliance with the study medication, condition of their health, and if they had visited other heath facilities and the medications they had taken. In addition they had a focused history and, obstetric examination using a standard clinical record form. At each visit the blood pressure of the women was measured using a mercury sphygmomanometer. Two blood pressure measurements were taken and an average was obtained. If a woman had a high blood pressure a mid-stream urine sample was collected for protein estimation using a dipstick. All the women received oral iron and folic acid supplements which they took daily and, de-worming tablets and intermittent presumptive treatment for malaria according to the Uganda Ministry of Health guidelines.

The women who developed hypertension were managed according to the protocol of the hospital.

### Outcomes

The primary end point was development of pre-eclampsia in the mother. The secondary outcomes were development of severe pre-eclamsia and gestational hypertension in the mother and, in the infant were low birth weight, still birth delivery and preterm delivery.

Pre-eclampsia was defined as described by the International Society for the Study of Hypertension in Pregnancy [29]. Under this classification, hypertension was defined as a blood pressure of ≥140/90 mmHg measured with a woman in a sitting position using a mercury sphygmomanometer. The blood pressure was repeated after 4 hrs. Significant proteinuria was taken as ≥2+ protein on dipstick of mid stream urine or catheter specimen urine if membranes were ruptured on two urine specimens 4 hrs or more apart. Pre-eclampsia was taken as hypertension with significant proteinuria, after 20 weeks gestation.

A woman was taken to have severe pre-eclampsia if she developed one or more of the following: a blood pressure of ≥160 mmHg systolic or ≥110 mmHg diastolic, ≥3+ protein by dipstick on two urine samples taken four hours or more apart, visual disturbances, frontal or any type of headache, epigastric or right upper quadrant pain, pulmonary oedema or cyanosis, abnormal liver function, low platelets, oliguria of less than 500 ml in 24 hours and fetal growth restriction [30].

Gestational hypertension was defined as hypertension which developed after 20 weeks of pregnancy in a woman who previously had a normal blood pressure and the blood pressure reverted to normal after delivery.

Low birth weight was defined as delivery of a baby whose birth weight was less than 2500 gm. A still birth was defined as delivery of a baby which died in uterus after 24 weeks of pregnancy. Preterm delivery was taken as delivery before 37 weeks of gestation.

### Sample size

We assumed that supplementation with vitamin C would lower the risk of pre-eclampsia by 50% compared to a placebo. We estimated the risk of pre-eclampsia to be 10% in the placebo arm based on the literature [31–33] and Mulago Hospital, Department of Obstetrics and Gynaecology annual reports [34]. Given these estimates a sample size of 932 women would give a power of 80% at 95% confidence level.

### Interim analyses and stopping guidelines

We performed one interim analysis when the accumulating data had accrued approximately half the estimated sample size according to O’Brien-Fleming boundaries (DeMets error-spending function) at a level α = 0.05 (two sided); the significance level for the final analysis was α = 0.0459. A standardized test statistic was calculated for the incidence of pre-eclampsia and all adverse effects based on accrued data. The Data Safety and Monitoring Board recommended continuation of the study.

### Randomization: sequence generation

An independent pharmacist allocated the active tablets or placebo tablets according to the computer generated randomization list. The study participants were given drugs every month until they delivered.

### Randomization: type

The randomization sequence was created using stata 12 software package in ratio of 1:1. Permutated block size of 6 and 8 were used and these were varied at random.

### Randomization: allocation concealment

The vitamin C and placebo tablets were identical in size, shape and colour. The study medications were packed in sealed white opaque bottles and were consecutively numbered for each woman according to the randomization schedule by a researcher who was not involved in the recruitment or clinical care.

### Randomization: implementation

The randomization was done by an independent statistician who was not involved in the study. Computer generated randomization codes were used to generate the randomization list which were sent to the pharmacy.

The women were allocated a study number by the research assistants who recorded the enrollment date and escorted the women to the pharmacy to receive the treatment assignment code and received the study medications.

### Blinding

The participants, the care providers and those assessing the outcomes were blinded to the study allocation.

### Similarity of interventions

Rene Industries Ltd. (Kireka, Kampala, Uganda) prepared vitamin C (ascorbic acid) and identical placebo tablets (microcrystalline cellulose). The tablets were identical in color, shape and size.

### Statistical methods

The Data were cleaned, coded and double entered using EPIDATA version 3.1 statistical package and transferred to STATA version 12 for analysis. Analyses were done using intention-to-treat principle: all randomly assigned groups were included in the group to which they were initially assigned. Women who were lost to follow-up were not included in the analyses of outcomes. Continuous variables were compared using a t-test and categorical variables between groups were compared using Chi squared test. Risks of the outcomes of interest were calculated and compared between the treatment and control arms. The results are presented as risk ratios with the corresponding 95% confidence intervals. Adjusted analyses were not done since the groups were comparable at baseline. This trial is registered as Pan African Clinical Trial, Registry PACTR 2012100000418271.

### Ethical approval

This study was approved by the Makerere University College of Health Sciences Ethics Committee, The Mulago Hospital Ethics Committee, The National Council for Science and Technology in Uganda and the Uganda National Drug Authority. The women gave written informed consent.

## Results

**Figure 1:**
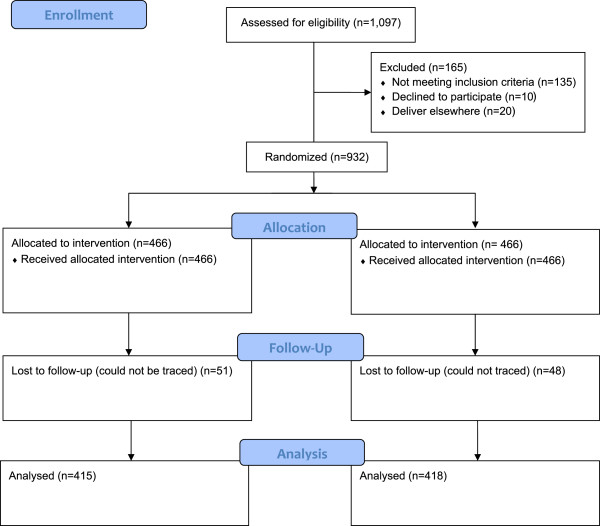
**Trial profile.**

We screened 1,097 women, of which 956 were eligible but 932 women were willing to participate in the study and were randomized (466(50%) to the vitamin C group and 466(50%) to the placebo group). The losses to follow-up were 51(10.9%) women in the vitamin group and 48(10.3%) women in the placebo group (Figure 1). Recruitment started in November 2011 and was completed in June 2012.

The enrollment and randomization of the women is as shown in Figure 1 below according to the flow chart suggested by the Consolidated Standards of Reporting Trials (CONSORT) [35].

### Recruitment

Eligible participants were recruited from November 2011 to June 2012. Participants attended the clinic at the time of randomization (baseline) and at four weekly intervals until January 2013.

### Baseline data

The baseline characteristics are shown in Table 1. There were no differences in the participants’ baseline characteristics (viz. age, educational level, marital status, parity, alcohol consumption, HIV status, smoking status, booking gestational age and booking blood pressure) at randomization.

**Table 1:** **Baseline characteristics of the participants**

Characteristic	Vitamin C (N = 466)	Placebo (N = 466)	P values
	n (%)	n (%)	
**Age group (years)**			0.77
≤19	91 (19.5)	98 (21.0)	
20-29	247 (53.0)	253 (54.3)	
30-34	79 (17.0)	69 (14.8)	
≥35	49 (10.5)	46 (9.9)	
**Marital status**			0.12
Married	403 (86.5)	386 (82.8)	
Single	63 (13.5)	80 (17.2)	
**Educational level**			0.41
Primary or less	186 (39.9)	174 (37.3)	
Secondary or above	280 (60.1)	292 (62.7)	
**Parity**			0.86
Primigravidae	135 (29.0)	129 (27.7)	
Gravida 2-3	187 (40.1)	186 (39.9)	
Gravida ≥4	144 (30.9)	151 (32.4)	
**Alcohol consumption**			0.8
Yes	41 (8.8)	39 (8.4)	
**HIV status**			0.42
Positive	39 (8.4)	46 (9.9)	
Negative	427 (91.6)	420 (90.1)	
**Smoking**			0.99
Yes	1 (0.2)	1 (0.2)	
*Booking gestational age	19.3 (SD 3.8)	19.7 (SD 4.0)	
*Booking systolic BP (mmHg)	113.9 (SD 12.8)	114.9 (SD 14.1)	
*Booking diastolic BP (mmHg)	67.5 (11.4)	68.1 (11.2)	
Hypertension in family			0.31
Yes	64 (13.7)	75 (16.1)	

*These are presented as means (standard deviations). BP: Blood pressure, HIV: Human Immunodeficiency Virus. For binary variable only yes category was reported.

### Numbers analyzed

The primary analysis was by intention-to-treat and patients were analyzed in the groups in which they were randomly assigned. Fifty one women in the vitamin group were lost to follow up, therefore 415 women were available for the analysis of outcomes; meanwhile 48 women were lost to follow up in the placebo group and 418 women were available for analysis of outcomes. Eight women in the vitamin group and 6 women in the placebo group had changed their residences; the rest of the women could not be traced by telephone. There were no differences in the demographic characteristics in the women who were lost to follow up (Table 2). There were no differences in the baseline characteristics between the women who were lost to follow up and the women who turned up.

**Table 2:** **Characteristics of women lost to follow up in the clinical trial**

Characteristic	Vitamin C N = 51	Placebo N = 48	P value
	n (%)	n (%)	
**Age group (years)**			0.4
≤19	17 (33.3)	21 (43.7)	
20-29	21 (41.2)	21 (43.7)	
30-34	6 (11.8)	3 (6.3)	
≥35	7 (13.7)	3 (6.3)	
**Marital status**			0.5
Married	37 (72.6)	32 (66.7)	
Single	14 (27.4)	16 (33.3)	
**Educational level**			0.6
Primary or less	25 (49)	26 (54)	
Secondary or above	26 (51)	22 (46)	
**Parity**			0.8
Primigravidae	24 (47.0)	25 (52.1)	
Gravid 2-3	13 (25.5)	10 (20.8)	
Gravid ≥4	14 (27.5)	13 (27.1)	
**Alcohol consumption**			0.3
Yes	2 (3.9)	4 (8.3)	
**HIV status**			0.7
Positive	4 (7.8)	3 (6.3)	
Negative	47 (92.2)	45 (93.7)	
**Hypertension in family**			0.6
Yes	7 (13.7)	8 (16.7)	

HIV: Human immunodeficiency virus.

Compliance was assessed as the percentage of tablets consumed over the total number of tablets supplied. Compliance was similar in both groups (median 85%).

### Outcomes

The primary outcome was the incidence of pre-eclampsia. Pre-eclampsia was 3.1% in the vitamin group and 4.1% in the placebo group (RR: 0.77; 95% CI 0.37-1.56) (Table 3). The secondary maternal outcomes were: severe pre-eclampsia 1.2% in the vitamin and 1.0% in the placebo group (RR: 1.25; 95% CI: 0.34-4.65) and gestational hypertension 7.7 in the vitamin and 11.5 in the placebo group (RR: 0.67; 95% CI: 0.43-1.03). Other maternal outcomes: ante partum haemorrhage, premature rupture of membranes and abruption placenta were similar in both groups (Table 3). There were two cases of eclampsia in the placebo group.

**Table 3:** **Maternal outcomes between the vitamin and placebo groups**

Characteristic	Vitamin C	Placebo	Risk ratio	P value
	N = 415	N = 418		
**Pre-eclampsia**				
Yes	13 (3.1)	17 (4.1)	0.77 (0.37-1.56)	0.4
Severe pre-eclampsia				
Yes	5 (1.2)	4 (1.0)	1.25 (0.34-4.65)	0.72
**Gestational hypertension**				
Yes	32 (7.7)	48 (11.5)	0.67 (0.43-1.03)	0.06
**Ante partum haemorrhage**				
Yes	7 (1.7)	9 (2.2)	0.78 (0.29-2.1)	0.6
**Premature rupture of membranes**				
Yes	15 (3.6)	19 (4.6)	0.79 (0.41-1.54)	0.5
**Abruption**				
Yes	1 (0.2)	2 (0.5)	0.5 (0.04-5.53)	0.6
**Delivery route**				
Vaginal	278 (67.0)	281 (67.2)	1.0 (0.82-1.22)	0.9
Caesarean	137 (33.0)	137 (32.8)		
**Twin delivery**				
Yes	14 (3.4)	11 (2.6)	1.28 (0.59-2.79)	0.5
**Eclampsia**				
**Yes**	0 (0)	2 (0.5)	0	

For binary variable, only the Yes category has been reported.

Overall, the incidence of pre-eclampsia was 3.6% (30/833). The incidence was 1.9% (4/215) among primigravidae, 2.3% (8/350) among gravida two to three, and 6.7% (18/268) among women who were gravida four or more (Table 4).

**Table 4:** **Incidence of pre-eclampsia in the different parities**

Characteristic	Pre-eclampsia	95% CI
**Parity**	**n (%)**	
Primegravidae	4 (1.8)	0.5-4.6
Gravida 2-3	8 (2.3)	0.9-4.6
Gravida ≥4	18 (6.7)	4.0-10.4
Total	30 (3.6)	

CI: Confidence Interval.

The secondary neonatal outcomes were low birth weight, still birth delivery and preterm delivery. The low birth weight rate was 11.1% in the vitamin group and 10.3% in the placebo group (RR: 1.07; 95% CI: 0.72-1.59), the still birth rate was 4.6% in vitamin group and 4.5% in the placebo group (RR: 1.01, 95% CI: 0.54-1.87) and preterm delivery was 11.3% in the vitamin C group and 12.2% in the placebo group (RR: 0.92; 95% CI: 0.63-1.34) There were no differences in the other neonatal outcomes between the groups (Table 5).

**Table 5:** **Neonatal outcomes between the vitamin and placebo groups**

Characteristic	Vitamin C (N = 415)	Placebo (N = 418)	Risk ratio	P value
	n (%)	n (%)		
**Birth weight**				0.7
<2500 gm	46 (11.1)	43 (10.3)	1.07 (0.72-1.59)	
≥2500 gm	369 (88.9)	375 (89.7)		
**Apgar score**				0.4
<7	41 (9.9)	35 (8.4)	1.17 (0.76-1.81)	
≥7	374 (90.1)	383 (91.6)		
**Admission to special care unit**				0.07
Yes	41 (9.9)	27 (6.5)	1.53 (0.95-2.43)	
**Still birth**				0.9
Yes	19 (4.6)	19 (4.5)	1.01 (0.54-1.87)	
**Early neonatal death**				0.4
Yes	7 (1.7)	10 (2.4)	0.71 (0.27-1.83)	
**Abortion**				0.9
Yes	9 (2.2)	9 (2.2)	1.01 (0.40-2.51)	
**Preterm delivery**				0.7
Yes	47 (11.3)	51 (12.2)	0.92 (0.63-1.34)	

For binary categories, only the yes category was reported.

## Discussion

This study was a randomized placebo controlled trial in which antioxidant vitamin C was given to healthy pregnant women daily from the second trimester until they delivered to reduce the risk of pre-eclampsia. Supplementation with high doses of vitamin C did not reduce the risk of pre-eclampsia. This is in contrast with an earlier study by Chappell and colleagues [24] which investigated the effect of 1000 mg of vitamin C and 400 IU of vitamin E on 239 women from 18-22 weeks gestation until delivery on the risk of pre-clamspia. The incidence of pre-eclampsia was 8% in the supplemented group versus 17% in the placebo group. However, in this study pre-eclampsia was a secondary outcome and the study was stopped early because the interim analysis indicated that there was significant improvement in the markers of endothelial activation which was the primary outcome and thus there were only 79 women in the vitamin group who completed the study versus 142 in the placebo arm. There could have been type I error since the sample size was small.

The other studies [22, 36] in which vitamin C was shown to be of benefit were epidemiological studies. Zhang and colleagues [36] conducted a case-control study in which women with a low intake of vitamin C were at an increased risk of pre-eclampsia. There could have been a recall bias by the women and the study design could not establish a cause-effect relationship. Similarly, Klemmensen and others [22] conducted a prospective population based cohort study in which women with a low dietary intake of vitamin C were found to have an increased risk of severe forms of pre-eclampsia. There could have been a misclassification of pre-eclampsia and other hypertensive diseases in pregnancy since the case ascertainment were a self report by the women. There could have also been a recall bias by the women on the food consumption and hence the evaluations of the vitamin C intake may not have been accurate.

However, the finding in our study is in agreement with what found by other researchers about use of antioxidants to prevent pre-eclampsia [25–27, 37]. Beazley and colleagues [25] studied the use of antioxidants among women at increased risk of pre-eclampsia. They did not find a difference in the incidence of pre-eclampsia among women who received antioxidants and placebo group. However, this study was terminated early because of lack of funding and the required sample size was not realized. Rumbold and colleagues [26] studied low risk women with a high nutritional status in which high dose vitamin C and vitamin E were given from second trimester until delivery. There was no reduction in pre-eclampsia between the two groups. In this study, the women’s baseline intake of vitamin supplements was above the recommended daily intake. In a study by Poston and collegues [37], women who were at an increased risk of pre-eclampsia were supplemented with high doses of vitamin C and vitamin E from the second trimester until they delivered. There was no reduction in the incidence of pre-eclampsia between the two groups.

Oxidative stress has been shown to play a role in the aetiology of pre-eclapmsia [15, 38, 39], and women with pre-eclampsia have increased markers of oxidative stress in the plasma and in the placenta [13]. However, results from this study and others indicate that use of high doses of antioxidants during pregnancy does not reduce the risk of pre-eclampsia. The explanation for this finding may be that while oxidative stress is present in women with pre-eclampsia, it may not play an important role in the pathophysiology of the disorder. It may also be possible that oxidative stress plays a role only in a subgroup of women with no appreciable effect on the general population. It also is possible that the women in our study had sufficient concentrations of vitamin C at trial entry. We based our assumptions on a previous study [40] in which most women were deficient of vitamin C. However, doses of vitamin C of 400 mg per day or higher result in plasma and tissue saturation and produces a steady-plasma concentration. When vitamin C doses of 500 mg per day or higher are given, the extra absorbed vitamin C is excreted to produce a steady plasma and tissue concretions [41]. Even in women who were at high risk of pre-eclampsia and nutritionally deficient [27], the antioxidants did not reduce the risk of pre-eclampsia.

In this paper the rate of severe pre-eclampsia was similar in intervention and placebo groups. This is similar to what was found in other studies among high risk women [24, 27, 37, 42] and women at low risk [43]. Similarly the rate of gestational hypertension did not differ between the intervention and placebo group. This is in agreement with was found by Villar and colleagues [27] in high risk women but is in contrast to what was found by Poston and colleagues [37] in high risk women and Roberts and colleagues [43] in low risk women in which women who took antioxidants were more likely to develop gestational hypertension than the controls. In addition, Poston and colleagues [37] and Rumbold and colleagues [26] found that women who took antioxidants were more likely to use antihypertensive therapy than controls. This was probably due to chance, but antioxidants may promote DNA oxidation through an interaction between vitamin C and metal ions [44, 45] leading lipid peroxidation and oxidative stress and, may produce mutagenic lesions in DNA [45].

In our study the low birth weight delivery rate was similar in the both the vitamin and placebo groups. This is in agreement with a study by Roberts and colleagues [43] in low risk women and Spinnato and colleagues [42] and, Villar and colleagues [27] in high risk women, in which the rates of low birth weight delivery was similar in the antioxidant and placebo group. But it differs from what was found by Poston and colleagues [37] in high risk women in which women who were supplemented with antioxidants were more likely to deliver low birth weight babies than the controls. This could be because the women who took supplements developed pre-eclampsia earlier and hence delivered at an earlier gestational age. An earlier study showed that vitamin C supplements given during pregnancy were associated with preterm delivery than controls [46] although this did not translate in poorer neonatal outcomes. However, this was more likely to be a chance finding as more women in this group had a previous history of miscarriage and ante partum haemorrhage.

In our study preterm delivery was similar between the intervention and control groups. This is similar to what was found in another study of intervention with antioxidants in high risk [27, 42] and low risk women [43]. The rate of still birth delivery was similar in intervention and control group. This similar to what was found by Roberts and colleagues [43] and Rumbold and colleagues [26] in low risk women and by Spinnato and colleagues [42] and by Villar and colleagues [27] in high risk women. However, post hoc analyses in the Rumbold and colleagues [26] study showed more still birth delivery in women who took antioxidants than the controls. However, the still birth rate was extremely high among women in this study. This could have been be due to delay of the women to reach the hospital after labour had started and institutional delay especially for women who needed caesarean section as the waiting time on average was 4-6 hours.

The other maternal outcomes (viz. ante partum haemorrhage, premature rupture of membranes, abruption placentae, mode of delivery) and neonatal outcomes (viz., apgar score, admission to the baby neonatal unit, early neonatal death) did not differ between the intervention and placebo groups.

### Limitations

This study was conducted in the national referral hospital and some findings may not be generalized to all pregnant women in Uganda. The incidence of pre-eclampsia was lower than was initially anticipated. This could have led to estimation of a smaller sample size. Therefore the results should be interpreted with caution as they may not be generalized to all pregnant women because of type II error. However, this study has enabled us to establish the effects of vitamin C supplementation on other pregnancy outcomes in Uganda.

## Conclusion

Supplementation with high dose vitamin C did not reduce the risk of pre-eclampsia nor did it reduce the risk of low birth weight delivery, still birth delivery and preterm delivery in low risk women with poor nutritional status in our institution. We do not recommend the use of high dose vitamin C in pregnancy to prevent pre-eclampsia or other adverse maternal and neonatal outcomes.
